# Renal function, serum sodium level, and outcomes in hospitalized systolic heart failure patients: An analysis of the COAST study

**DOI:** 10.1097/MD.0000000000003898

**Published:** 2016-06-24

**Authors:** Jin Joo Park, In-Ho Chae, Dong-Ju Choi, Seok-Min Kang, Byung-Su Yoo, Juey-Jen Hwang, Shing-Jong Lin, Ming-Shien Wen, Jian Zhang, Junbo Ge

**Affiliations:** a Cardiovascular Center, Seoul National University Bundang Hospital, Seongnam, South Korea; b Severance Hospital, Yonsei University Health System, Seoul, Korea; c Yonsei University Wonju College of Medicine, Wonju Christian Hospital, Wonju, South Korea; d National Taiwan University Hospital, Taipei City, Taiwan; e Taipei Veterans General Hospital, Taipei City, Taiwan; f Chang Gung Memorial Hospital, Linkou, Taiwan; g Fuwai Hospital, Beijing, China; h Zhongshan Hospital, Shanghai, China.

**Keywords:** hospitalized heart failure, low sodium level, predictor of clinical outcomes, renal impairment

## Abstract

Both renal function and serum sodium level are well-known prognostic markers in heart failure (HF) patients. We investigated the prognostic value of the renal impairment (RI) stratified by the serum sodium level in systolic HF patients.

The Clinical Characteristics and Outcomes in Relation with Serum Sodium Level in Asian Patients Hospitalized for Heart Failure (the COAST) Study enrolled hospitalized systolic HF patients (ejection fraction<45%) in South Korea, Taiwan, and China. Twelve-month mortality was stratified according to the renal function and serum sodium level.

Of 1462 enrolled patients, 716 patients (49%) had RI (GFR<60 mL/min/1.73 m^2^), and they had higher 12-month mortality than those without RI (22.8% vs. 10.9%, *P*<0.001). Furthermore, 676 patients (46%) had low sodium level defined as Na<median, that was, 139 mmol/L. The mortality rate was lowest in patients with normal renal function and high sodium level (7.4%), but highest in those with RI and low sodium level (26.1%) (*P*<0.001). Patients with both RI and low sodium level had a 3.8-times increased hazard for 12-month mortality (HR 3.80, 95% CI 2.06–7.05), whereas the low sodium level (HR, 2.95; 95% CI, 1.51–5.75) and RI (HR 3.08; 95% CI, 1.63–5.82) had similar hazard, suggesting that they might be equivalent risk factors.

In hospitalized Asian HF-patients both RI and low sodium level are independent risk factors. Patients with both RI and low serum sodium level are at the highest risk and may require meticulous medical care.

## 1 Introduction

Hospitalizations for acute heart failure (HF) are increasing. Among various risk factors for poor clinical outcomes, a low serum sodium level is relatively common and associated with increased mortality among hospitalized HF patients.^[1–3]^ There are numerous interactions between heart and kidney. Patients with kidney disease have an increased risk for cardiovascular events,^[4]^ and *vice-versa.* More than half of the HF patients show renal impairment (RI),^[5–7]^ and HF-patients with RI have excess mortality.^[8,9]^

Thus both RI and low sodium levels are well-known risk factors for adverse outcomes in HF patients, and those patients confer a group of patients with notably high disease severity. However, the predictive value of RI complicated by a low sodium level has not been evaluated in hospitalized HF patients. Clarifying the risk could provide more precise risk stratification and the development of optimal therapeutic strategies in these patients.

In this multinational, multicenter study, we aim to clarify the magnitude of risk of RI and low sodium level contributing to outcomes in Asian hospitalized systolic HF patients.

## 2 Materials and methods

An Observational Study on the Clinical Outcomes in Relation with Serum Sodium Level in Asian Patients Hospitalized for Heart Failure (COAST) is a multinational, multicenter registry. The design and main outcomes have been previously reported elsewhere.^[10]^ In brief, we included 1650 HF patients with an age of 18 years or older and a left ventricular ejection fraction (LV EF) of <45% who were hospitalized for acute HF at 8 centers in Korea, Taiwan, and China since January 2009. Patients’ data on index admission, discharge, and 1-year follow-up were collected at each site by trained study coordinators and audit was performed by the COAST Registry Steering Committee. In this substudy, we enrolled patients in whom glomerular filtration rate (GFR) could be calculated. The study protocol was approved by the institutional review board (IRB) or ethics committee at each participating hospital, and the patient's informed consent was waived by the IRB of each participating center. The study complied with Declaration of Helsinki.

The serum sodium and creatinine levels were measured at hospital admission. The low serum sodium level was defined as serum sodium less than the median, which was 139 mmol/L. Using the median as a cutoff value allowed that the comparing groups had a similar sample size. The estimated glomerular filtration rate (eGFR) was calculated from creatinine values using the Modified Diet in Renal Disease (MDRD) formula.^[11]^ Patients were assigned a kidney function stage using National Kidney Foundation cutoffs for degrees of renal failure: stage I (normal; GFR > 90 mL/min/1.73 m^2^); stage II (mild damage; GFR 60–89 mL/min/1.73 m^2^); stage III (moderate damage; GFR 30–59 mL/min/1.73 m^2^); stage IV (severe damage; GFR 15–29 mL/min/1.73 m^2^); or stage V (kidney failure; GFR <15 mL/min/1.73 m^2^). We defined renal impairment as GFR <60 mL/min/1.73 m^2^.

The primary endpoint was the 12-month mortality according to the renal function and serum sodium levels. The secondary endpoints included the mortality rates at the index admission, 12-month rehospitalization rate, and the composite of mortality and/or rehospitalization.

Categorical variables were presented as numbers and frequencies and continuous variables as mean ± standard deviation. For comparison between groups, the chi-square test was used for categorical variables. The Wilcoxon rank-sum test was used for comparisons of serum sodium with continuous measures.

A Cox-proportional hazard regression analysis was applied to identify the independent predictors of 12-month mortality after adjustment for significant covariates. Receiver operating characteristics (ROC) curve analysis was used to determine the optimal cut-offs of the risk factors. All data were analyzed using SAS^®^ Version 9 (SAS Institute, Cary, NC) and SPSS version 17 (SPSS Inc., Chicago, IL). The statistical analysis was performed by professional statistical counsel.

## 3 Results

Of 1470 patients enrolled in the COAST main study, GFR could not be calculated in 8 patients, so that the data of 1462 patients were available for the current analysis. GFR was unimodally distributed at admission (median of 60 mL/min/1.73 m^2^ and interquartile range, 45–78 mL/min/1.73 m^2^). So was the distribution of serum sodium level at admission (median, 139 mmol/L and IQR, 136 – 141 mmol/L). The distribution of kidney stages was 14.4%, 36.6%, 34.6%, 9.7%, and 4.7% for stage I, II, III, IV, and V, respectively.

Almost half of the patients (716 patients, 49%) had RI, in contrast only 11.9% of the patients reported as having “chronic kidney disease” at the hospital admission. Table 1 shows the demographic and baseline characteristics of the patients according to the renal function and serum sodium level. Patients with RI had more adverse clinical characteristics, such as older age, higher frequency of diabetes mellitus, hypertension, dyslipidemia, and advanced New York Heart Association (NYHA) Class. Patients with RI had higher prevalence of hyponatremia, but lower frequency of prescription of rennin–angiotensin–aldosterone system (RAAS) inhibitor, beta blocker, and mineralocorticoid antagonist.

**Table 1:**
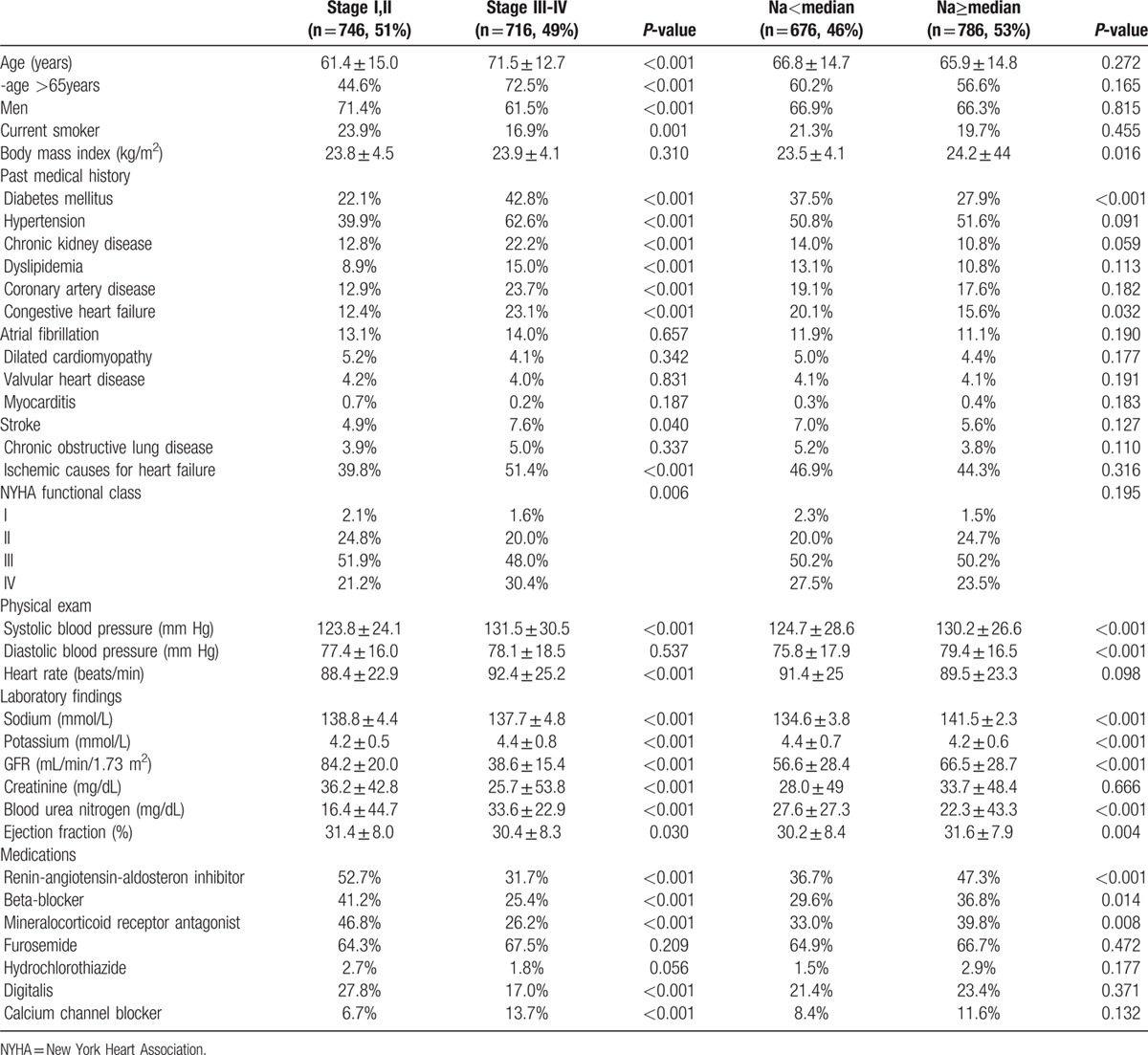
Baseline characteristics according to renal function.

**Figure 1:**
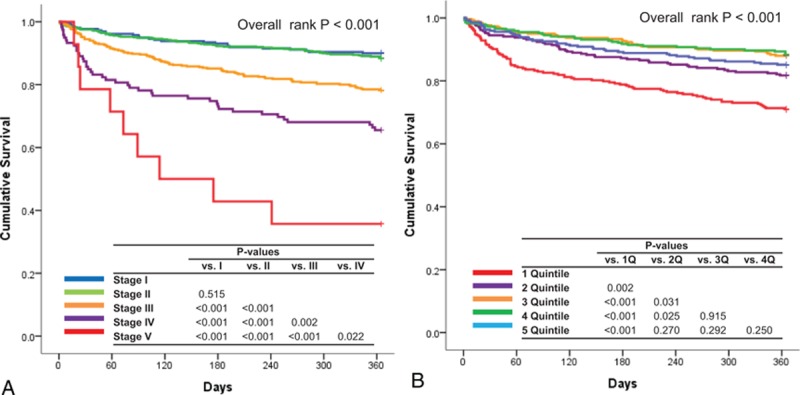
Clinical outcomes according to the renal function and serum sodium level. 12-month mortality according to the renal function (A), and serum sodium level quintiles (B).

During a 12-month clinical follow-up, 244 patients (16.7%) died. Patients who died had lower GFR (51.4 ± 29.7 vs. 64.0 ± 28.4 mL/min/1.73 m^2^, *P* < 0.001) and serum sodium level (136.7 ± 5.7 vs. 138.6 ± 4.3 mmol/L, *P* < 0.001) than those who survived. When stratifying according to the kidney stages, the 12-month mortality rate was 11.0%, 10.9%, 19.4%, 32.1%, and 31.6% for stage I,II, III, IV, and V, respectively (*P* < 0.001, linear-by-linear association, *P* < 0.001). In Kaplan–Meier Survival analysis, there was no difference in the mortality rate between kidney stage I and stage II, whereas the mortality increased with worsening renal function (Fig. 1A). Similarly, when stratifying the patients according to the quintiles of the serum sodium level, patients in 1st and 2nd quintiles had a higher 12-month mortality, whereas there was no difference between 3rd to 5th quintile (Fig. 1B).

**Figure 2:**
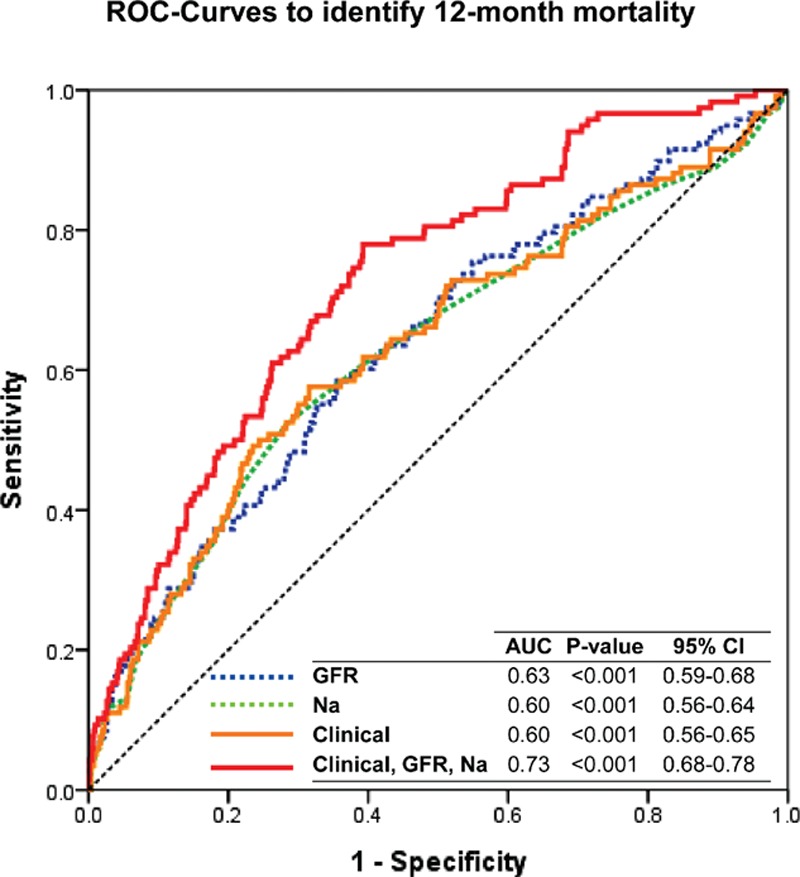
Receiver-operating characteristics curve analysis. The best cutoff value of GFR to predict the 12-month mortality was 60 mL/min/1.73 m^2^ with a sensitivity, a specificity, and an area under the curve (AUC) of 0.56, 0.68, and 0.63 (95% confidence interval [CI], 0.59–0.68; *P* < 0.001), respectively. The best cutoff value of serum sodium level was 138 mmol/L with a sensitivity, a specificity, and an AUC of 0.67, 0.51, and 0.60 (95% CI 0.56–0.64; *P* < 0.001), respectively. AUC = area under the curve, CI = confidence interval, GFR = glomerular filtration rate.

In ROC curve analysis, the best cutoff value of GFR to predict the 12-month mortality was 60 mL/min/1.73 m^2^ with a sensitivity, a specificity, and an area under the curve (AUC) of 0.56, 0.68, and 0.63 (95% confidence interval [CI], 0.59–0.68; *P* < 0.001), respectively. As for the serum sodium level, the best cutoff value was 138 mmol/L with a sensitivity, a specificity, and an AUC of 0.67, 0.51, and 0.60 (95% CI 0.56–0.64; *P* < 0.001), respectively (Fig. 2).

The 12-month mortality rate was higher in the RI group than in those without (22.8% vs. 10.9%, *P* < 0.001). After adjustment, RI was associated with a 40% increase in 12-month mortality (adjusted HR, 1.40, 95% CI 1.20–1.63). In total, 676 patients (46%) had a low serum sodium level. The clinical outcomes including in-hospital death, 12-month mortality, 12-month rehospitalization, and 12-month composite of mortality and rehospitalization were higher in patients with RI and low sodium level (Table 2).

**Table 2:**
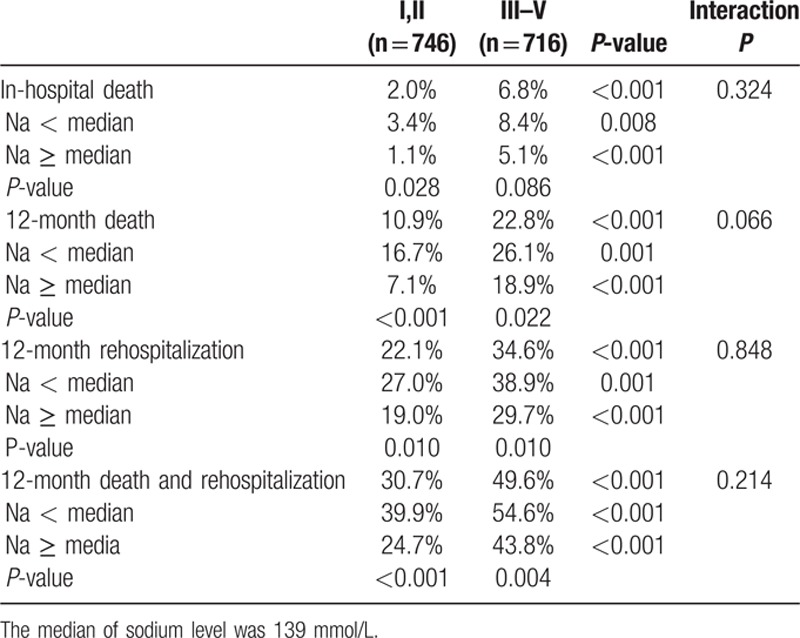
Clinical outcomes according to renal function and serum sodium.

**Figure 3:**
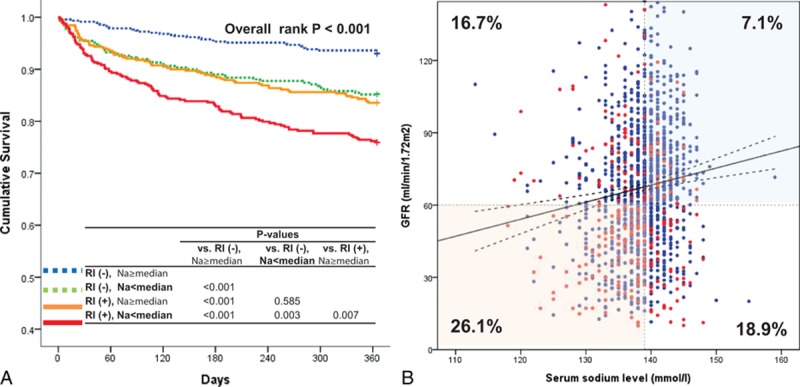
12-month mortality according to renal function and serum sodium level. The 12-month mortality rate was lowest in patients with normal renal function and high sodium level but highest in patients with renal impairment (RI) and low sodium level, whereas there was no difference between patients with normal renal function and low sodium level and those with RI and high sodium level (**A**). There was a week correlation between GFR and serum sodium level (Pearson's *r* = 0.115, *P* < 0.001) (B). GFR = glomerular filtration rate, RI = renal impairment.

There was only a week correlation between GFR and serum sodium level (Pearson's *r* = 0.115, *P* < 0.001). When classifying the patients according to renal function and serum sodium level, the 12-month mortality rate was lowest in patients with normal renal function and high sodium level (7.1%), but highest in patients with RI and low sodium level (26.1%) (log-rank *P* < 0.001), whereas there was no difference between patients with normal renal function and low sodium level (16.7%) and those with RI and high sodium level (18.9%) (log-rank *P* = 0.585) (Fig. 3). In the Cox-proportional hazard regression analysis, patients with both RI and low sodium level had a 3.8-times increased hazard for 12-month mortality (HR, 3.80; 95% CI, 2.06–7.05), whereas a low sodium level (HR, 2.95; 95% CI, 1.51–5.75) and RI (HR 3.08; 95% CI, 1.63–5.82) had similar hazard ratio, suggesting they might be equivalent risk factors (Table 3).

**Table 3:**
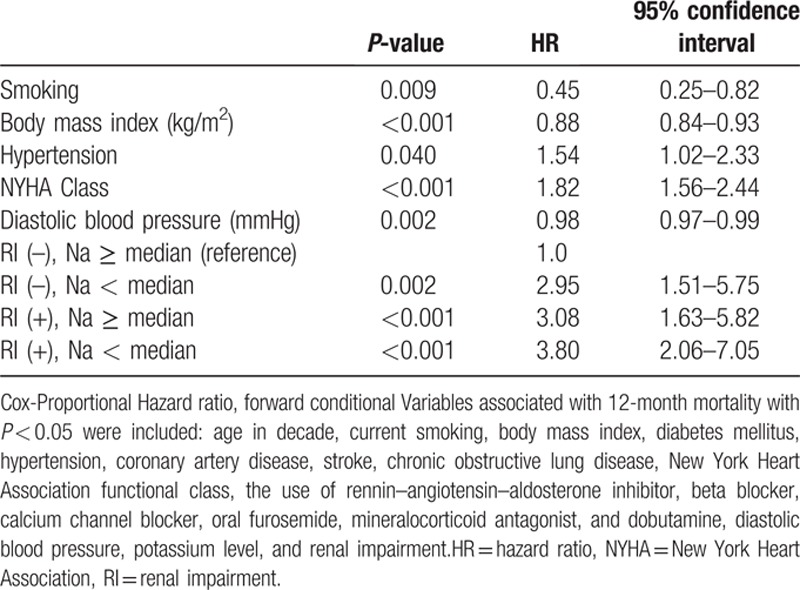
Independent predictor of 12-month death.

## 4 Discussion

In this study, we showed that in hospitalized HF patients the 12-month mortality increased as renal function and serum sodium level decreased. Half of the patients had RI and those with the low serum sodium level were at the highest risk for 12-month mortality. There existed a very weak correlation between renal function and serum sodium level. Both RI and low sodium level were independent predictors of worse clinical outcomes with similar effect size, suggesting both may be equivalent risk factors. To the best of our knowledge, this is the first study which investigated the prognostic value of RI stratified by the serum sodium level in hospitalized HF patients.

As HF is a disease of the elderly, many of the HF patients have other accompanying comorbidities. Among them, RI frequently coexists with HF.^[7,12]^ In this study, only 18% of the patients had normal renal function defined as Kidney Stage I, whereas the majority of the patients had some degree of renal dysfunction. This is similar to the study result, where only 17% of HF patients had creatinine clearance >90 mL/min/1.73 m^2^.^[13]^ Thus, renal dysfunction is the rule rather than the exception in hospitalized HF patients. National Kidney Foundation defines mild renal dysfunction with a reduction of GFR <90 mL/min/1.73 m^2^ (stage II). We demonstrated that there was no difference in 12-month mortality between kidney stage I and II, whereas the 12-month mortality increased with worsening kidney stages (III–V). This finding indicates a threshold effect of GFR, suggesting no risk increase above this threshold.^[14]^ It also implies a dose–response relationship below the threshold and may be an indirect evidence for the causality between renal function and clinical outcomes.

The low serum sodium level has been reported as a risk factor for worse clinical outcomes.^[1–3]^ We showed that there was a very weak correlation between GFR and serum sodium level. Both RI and low sodium level have been associated with advanced HF. Interestingly, RI (HR, 3.08; 95% CI, 1.63–5.82) and low sodium level (HR, 2.95; 95% CI, 1.51–5.75) had similar hazard ratio, suggesting that they might be equivalent risk factors (log-rank *P* = 0.585). More importantly, patients with both RI and low sodium level confer a group of patients at the highest risk for 12-month mortality (HR, 3.80; 95% CI, 2.06–7.05).

As for mechanistic explanation, both the kidney and heart contribute to the maintenance of hemodynamic stability, and each dysfunctional organ has the ability to initiate and perpetuate disease in other organ through common hemodynamic, neurohormonal, and inflammatory feedback pathway: upregulation of the RAAS, enhancement of sympathetic nerve stimulation, and increase in proinflammatory factors.^[15]^ It is not clear, whether RI is a marker for or a mediator of worsening HF. If it was a “mediator,” cardio-renal pathophysiologic pathways could be an important target for preventing worsening HF.

Similarly, both neuro-humoral overactivation and nonosmotic release of vasopressin due to decreased effective circulating volume contribute to development of hyponatremia.^[16]^ Consequently, hyponatremia is observed in more advanced HF patients. The role of hyponatremia as a mediator is still controversial; the correction of hyponatremia during hospital admission was not associated with improved outcomes in 2 retrospective cohort studies,^[10,17]^ whereas its correction with AVP antagonist showed improved outcomes in a post-hoc analysis of a randomized clinical trial.^[18]^

Despite high prevalence of RI, only 12% of the patients reported having chronic kidney disease in their history taking. Considering the substantial impact of RI in HF patients, this discrepancy mandates routine measurement of GFR in all HF patients and to incorporate renal function into risk stratification.

The overall prescription rate of RAAS inhibitor, beta blocker, and mineralocorticoid receptor antagonist was 67%, 57%, and 55% at the hospital discharge. However, there exist a clear trend for underprescription in patients with RI and low sodium level. RAAS inhibition has been the mainstay of HF-therapy and has proven reno-protective effect in patients with hypertension and diabetic nephropathy.^[19]^ Furthermore, RAAS inhibition improves survival in patients with systolic heart failure,^[20–22]^ as well as in those with severe RI.^[23,24]^ Therefore, despite the transient GFR reduction in HF-patients with underlying renal dysfunction,^[13,25,26]^ RAAS inhibitors are still recommended and should be used unless contraindicated.

With regard to diuretics, the usage of hydrochlorothiazide was higher in patients with normal renal function than in those with RI (2.7% vs. 1.8%, *P* = 0.056) with a marginal significance. According to K/DOQI clinical practice guidelines, thiazide diuretics are recommended in patients with GFR ≥30 mL/min/1.73 m^2^ because of their insufficient potency at reduced GFR.^[27]^ Loop diuretics have lower hyponatremic risk than thiazide-type diuretics because they inhibit sodium chloride reabsorption in the thick ascending limb of the loop of Henle, leading to impaired generation of the hyperosmotic gradient in the medullary interstitium. Consequently, the responsiveness to antidiuretic hormone is reduced. The opposite is for thiazide-type diuretics. In our study group, the usage of thiazide diuretics did not differ between patients with high and those with low serum sodium level, suggesting that causes other than diuretics may be responsible for the different serum sodium levels.

The main limitation of the present study is the COAST study was not a prospective, randomized trial. Although we tried to adjust for significant clinical variables, unmeasured confounding factors may have biased the study result. Furthermore, a single measurement would not fully describe the patient's condition in this highly vulnerable and dynamic phase of acute decompensation. Serial measurement would better describe the patient's reserve capacity to recover from acute decompensation.

In conclusion, hospitalized HF patients both RI and low serum sodium level are independent predictors of worse clinical outcomes. Especially, RI patients with low serum sodium level are at the highest risk and need meticulous medical care in hospitalized HF patients.
